# Role of Dehydrodiferulates in Maize Resistance to Pests and Diseases

**DOI:** 10.3390/ijms11020691

**Published:** 2010-02-09

**Authors:** Rogelio Santiago, Rosa A. Malvar

**Affiliations:** Misión Biológica de Galicia (CSIC), Apartado 28, E-36080, Pontevedra (PC 36143), Spain

**Keywords:** plant cell walls, ferulic acid, dehydrodimers, maize resistance-mechanism, corn borer, maize weevil, *Gibberella* ear, stalk rot

## Abstract

Phenolic esters have attracted considerable interest due to the potential they offer for peroxidase catalysed cross-linking of cell wall polysaccharides. Particularly, feruloyl residues undergo radical coupling reactions that result in cross-linking (intra-/intermolecular) between polysaccharides, between polysaccharides and lignin and, between polysaccharides and proteins. This review addresses for the first time different studies in which it is established that cross-linking by dehydrodiferulates contributes to maize’s defences to pests and diseases. Dehydrodiferulate cross-links are involved in maize defence mechanisms against insects such as the European, Mediterranean, and tropical corn borers and, storage pest as the maize weevil. In addition, cross-links are also discussed to be involved in genetic resistance of maize to fungus diseases as *Gibberella* ear and stalk rot. Resistance against insects and fungus attending dehydrodiferulates could go hand in hand. Quantitative trait loci mapping for these cell wall components could be a useful tool for enhancing resistance to pest and diseases in future breeding programs.

## 1. Introduction

**Figure 1.**
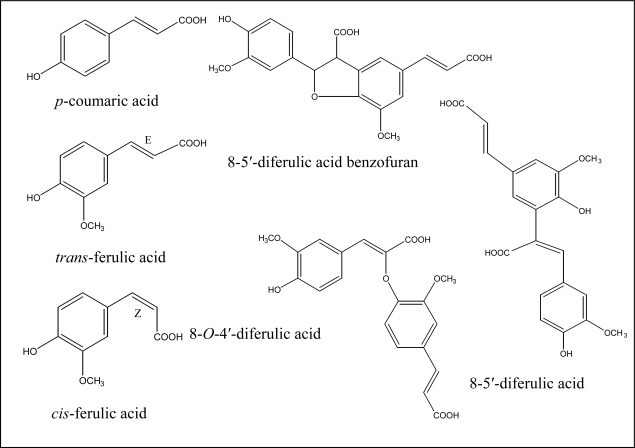
Chemical structures of cell wall phenylpropanoids identified in diverse maize tissues (modified from Santiago *et al.* [56]).

In the Poaceae family (grasses and cereals) the primary cell wall is built of a skeleton of cellulosic microfibrils embedded in a matrix composed mainly of hemicelluloses (arabinoxylans, xyloglucan and, in some tissues, mixed-linked glucans) and smaller amounts of pectins and glycoproteins [1]. In addition, grasses have unusually high concentrations of ferulic and *p*-coumaric acids ester-linked to cell wall polymers (Figure 1) [2]. Depending on the tissue and its stage of development, cell walls in C4 grasses tend to have higher levels of hydroxycinnamic acids than C3 grasses; as C4, maize and sorghum cell walls can contain up to 4% ferulates (monomers plus dimers) and up to 3% *p*-coumarate [3,4]. *p*-Coumarate is mainly esterified to the γ–position of phenylpropanoid sidechains of S units in lignin [5–7]. Although very small quantities of *p*-coumarate are esterified to arabinoxylans in immature tissues, most *p*-coumarate accretion occurs in tandem with lignification [8,9], making *p*-coumarate accumulation a convenient indicator of lignin deposition. Ferulates are intracellularly esterified to the C5-hydroxyl of α-l-arabinose sidechains of xylans and deposited into primary and secondary walls of a variety of grass tissues [10–12]. During cell wall deposition and lignification, xylans are cross-linked by peroxidase-mediated coupling of ferulate monomers into a complex array of dimers and trimers and by extensive copolymerization of these ferulates into lignin (Figure 1) [13].

## 2. Cell Wall Cross-Linking by Dehydrodiferulates

**Figure 2.**
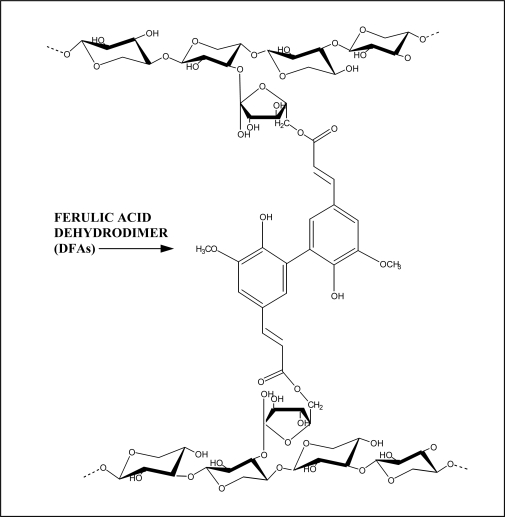
Cell wall cross-linking between two arabinoxylans chains mediated by dehydrodiferulates (DFAs) (from Santiago *et al.* [57]).

The structural and functional roles of plant cell walls are controlled by the composition and organization of individual wall components. Cross-linking of cell wall components is expected to have a marked influence on numerous wall properties such as accessibility, extensibility, plasticity, digestibility, and adherence. Phenolic esters have attracted considerable interest due to the potential they provide for peroxidase catalysed cross-linking of cell wall polysaccharides [14,15]. Dimers of ferulic and *p*-coumaric acids can be generated enzymatically by peroxidase (5,5’-diferulic acid) or through photochemical reactions (truxillic and trucinic acids) [16]. In particular, feruloyl residues undergo radical coupling reactions that result in cross-linking between polysaccharides (intra-/intermolecularly) (Figure 2) [4,17], between polysaccharides and lignin [18,19] and, between polysaccharides and proteins [20].

Formation of oxidatively coupled ferulate dimers was first described in 1971 [21]. Since then, a diverse range of dehydrodiferulates have been detected in various plant materials and were characterized as 8-5′, 5-5′, 8-O-4′, 8-8′, and 4-O-5′ coupled dehydrodimers [22–30]. In addition, certain specific isomers of triferulic acid [31–35] and tetraferulic acid [33] have been isolated and structurally elucidated from primary cell walls in grass and cereal species, particularly from maize bran.

Cross-linking of wall polysaccharides is of considerable interest in food chemistry, food technology and nutritional sciences, but also in neighbouring disciplines like agricultural chemistry and plant physiology [36]. Ferulate dimers have been considered responsible for phenomena such as terminating the expansion of cell wall growth [24], cell stiffening [37], cell wall adhesion influencing the cripness and softening fruits and vegetables [38–41], gelling properties of pectins and arabinoxylans [39,42,43], insolubility of cereal dietary fibers [30], and limited cell wall degradability by ruminants [3,13,44,45]. Cross-linking by dehydrodiferulates also contributes to plants’ defences to pest and diseases as revised in detail in the current review.

## 3. Role of Dehydrodiferulates Compounds in Maize Resistance to Pests

### 3.1. Corn Borer Resistance

Lepidopteran stalk boring larvae cause significant economic losses to maize (*Zea mays* L.) production throughout the world. One of the major corn borer pests is the European corn borer (ECB) [*Ostrinia nubilalis* (Hübner)] [46]. This pest reduces maize yield not only damaging kernels by direct feeding, but also tunneling stalks which cause plant lodging at harvest. ECB also acts as a vector for several *Fusarium* stalk-rot fungi which lead to a further destruction of the stalk. Infestation begins at an early phenological stage of the maize plant. First generation larvae fed on the whorl leaf tissues of juvenile plants, while subsequent generations attack maize at its reproductive stage. The main importance is on later generations larvae that feed on the stalk.

In studies of borer resistance, the content of isomers of dehydrodiferulic acids (DFAs) in corn leaves was highly and negatively correlated across genotypes of maize with field leaf damage caused by ECB [47]. In a later study, those authors suggest that early cell wall fortification through phenolic cross-linking of hemicellulose by diferulic acid increases leaf tissue toughness, and therefore, decreases neonate larvae penetration into leaf tissues [48]. Additional confirmation was provided by the observation that DFA content of maize increased in different tissues over cycles of selection for borer resistance in the Iowa stiff stalk synthetic BS9 [49]. High fibre content alone may increase the bulk density of the tissue, but in presence of phenolic-carbohydrate complexes, fibre strength may increase, providing a tougher physical barrier to restrict insect penetration and render nutrients within the tissues less accessible [49]. Besides, in the same study, both DFA and *p*-coumaric acids were negatively related with damage parameters for rind, node and pith tissue [49].

Ramputh [50] extended previous research with the observation that resistance to important tropical and subtropical stem borers could be sensitive to early cell wall fortification by phenolic cross-linking. Host plant resistance in maize to the southwestern corn borer (*Diatraea grandiosella* Dyar) and the sugarcane borer [*Diatraea saccharalis* (Fabricius)] was investigated in relation to bound phenolics in the leaf cell wall. In that study, dehydrodiferulic acids were found to be significantly correlated to tissue toughness and significant negative correlations were found between DFA and leaf feeding damage by both insects.

The oxidative potential of H_2_O_2_ to contribute to cell wall strengthening could retard insect feeding through the oxidative cross linking of specific cell wall structural proteins [51], polymerization of coniferyl alcohol affecting lignin formation [52] and cell wall peroxidase mediated cross linking of hemicellulose microfibrils with diferulic acid DFA [53]. A molecular transformation of maize was employed to increase cell wall hydrogen peroxide in order to enhance DFA formation and European corn borer resistance [54]. Corn transformed with a wheat oxalate oxidase gene (germin) under the control of the rice promoter elements (pActOXO) expressed the enzyme activity constitutively. Field testing showed that the transgenic maize exhibited more resistance to ECB than the non-transgenic counterpart. However, the transformation did not increase DFA formation as predicted, and resistance was associated with the direct effects of H_2_O_2_ on insect physiology. In a subsequent study was found that transgenic oxalate oxidase elicits defence responses by generation of H_2_O_2_, increasing soluble phenolics contents, and activating jasmonic acid signaling [55].

Recent studies evaluated the putative role of dehydrodiferulate contents in maize pith and leaf-sheaths in the resistance against the Mediterranean corn borer (MCB) (*Sesamia nonagrioides* Lef.) [56–58]. Three hydroxycinnamates, *p*-coumaric, *trans*-ferulic, and *cis*-ferulic acids, and three dehydrodiferulates, 8-5′, 8-O-4′, and 8-5′ b (benzofuran form), were identified [56]. The amount of all these compounds in the pith was correlated with the resistance level in the genotypes, with the resistant inbreds having the highest concentrations [56]. In addition, significant negative correlations were found between larvae weight reared on leaf-sheaths and diferulic acid content for six genotypes of the seven evaluated [57].

The genotypes used in those studies were a limited number of inbred lines with diverse background and perhaps exhibiting different mechanisms of defence. Therefore, the real relationship between DFA content and resistance was not established because it could be biased by background differences among genotypes. In order to remove this bias, several cycles of selection for MCB resistance derived from the synthetic population EPS12 were evaluated for cell-wall phenolic concentrations in the pith tissues [58]. In this sense, higher concentrations of total DFAs were associated with less tunnel length and number of larvae per stem [58]. Santiago and co-workers [58] showed new and concrete evidence that the cell-wall bound phenolics could have a significant role in the resistance to the MCB, although current development of divergent recurrent selection cycles maximizing differences for DFA concentrations will definitively clarify the role of DFAs in the resistance to the Mediterranean corn borer larvae. In 2007, the divergent selection for total DFAs content in the pith was initiated from the F_2_ derived from the cross of two inbred lines, CO125 (high DFA content) and PB130 (low DFA content).

### 3.2. Maize Weevil Resistance

The maize weevil (MW) (*Sitophilus zeamais* Motsch.) is a destructive insect feeding on stored maize throughout the world [59]. Subsistence farmers in tropical and subtropical agroecosystems often experience grain damage exceeding 30% during on-farm storage [60]. Various biochemical and physical characteristics have been identified as mechanisms of kernel resistance to MW [61–65]. Phenolic acids have been studied extensively as biochemical components correlated with resistance and found to act in two ways: through mechanical resistance (cell wall bound hydroxycinnamic acids) and antibiosis (phenolic acid amides) in the pericarp and aleurone layer, respectively [65]. Arnason and coworkers [64] has demonstrated that MW resistant genotypes have higher concentrations of total DFAs in the whole kernel. These dimers probably contribute to the observed correlations between phenolic acid content and grain hardness [65,66].

To understand the importance of dehydrodiferulates in MW resistance, a detailed analysis of DFAs in the pericarp was conducted by García-Lara and co-workers [67]. The analysis revealed the presence of three major simple phenolic acids (*p*-coumaric acid, *cis*-ferulic acid and *trans*-ferulic acid) and four isomers of DFAs (8-5′ DFA, 5-5′ DFA, 8-O-4′ DFA, 8-5′b DFA). In that study, DFA accounted for 8% of the total phenolic acid content, with resistant genotypes having a three-fold higher level than susceptible genotypes. On the basis of step-wise regression models, 5-5′ DFA, 8-O-4′ DFA, *p*-coumaric acid, and *trans*-ferulic acid were the most important phenolic acids in explaining phenotypic variance for MW resistance. Besides, weevil susceptibility was negatively correlated with total DFAs. In conclusion, maize genotypes with elevated levels of cell wall cross-linking components in the pericarp were more resistant to MW [67].

## 4. Role of Dehydrodiferulates Compounds in Maize Resistance to Fungal Diseases

### 4.1. Gibberella Ear Rot Resistance

*Fusarium graminearum* Schwabe, [teleomorph *Gibberella zeae* (Schwein.) Petch], is responsible for one of the major fungal diseases occurring sporadically in cool and temperate maize cultivation areas [68]. This disease known as *Gibberella* ear rot, is a cause of great concern because of the ability of this mold to produce mycotoxins, such as trichothecenes and zearalenone that affect grain quality and also represent a potential risk for livestock and human health [69–71].

Previous studies on maize indicate that phenolic compounds such as inducible flavones in silks [72] and structural phenolics in kernels [73,74] may be involved in maize resistance to ear rot. Attending to these results, Bily and co-workers [75] determined the concentrations of dehydrodimers of ferulic acid in the pericarp and aleurone tissues of some maize genotypes of varying susceptibility. Significant negative correlations were found between disease severity and total DFAs content, and even stronger negative correlations were observed between total DFA and ergosterol, a fungal component of the cell membrane which is an indicator of fungal biomass in infected plant tissues [75]. DFAs are known to stabilize the wall at the early stage of plant development [76]. It is also known that DFAs impede *in vitro* cell-wall degradation by fungal hydrolases, such as *Aspergillus* esterases [77]. In Bily’s study [75], initial penetration in the ears was mechanically facilitated, but mycelial progression on intact neighboring grains was reduced in maize genotypes with high levels of DFAs. Besides, *F. graminearum*, as many other pathogenic fungi, has a large spectrum of extracellular enzymatic activities [78,79], and the activity of fungal esterases can release free forms of ferulic ester from maize tissues. Once released, free ferulate may inhibit the ability of *Fusarium* to produce mycotoxins [80].

### 4.2. Gibberella Stalk Rot Resistance

*Fusarium graminearum* is only one of many species responsible for stalk rot, but is considered to possess the highest pathogenicity (the ability to cause disease) and aggressiveness (the amount of disease caused) [81,82]. These fungal organisms decay pith tissue in the lower stalk internodes, which can often result in premature plant death, poor kernel filling and/or lodging. Besides, trichothecenes such as deoxynivalenol (DON) is water soluble, and translocation in the phloem as a bulk flow of solution is assumed to occur [81,83]. DON produced in the stalk or ear of maize can be found in tissues not invaded by *F. graminearum* [84,85].

Phenolic compounds have been shown to inhibit *in vitro* growth of several fungal genera [86,87]; significant inhibitory effects were reported for *Fusarium* species [73,88,89]. In addition, evidence strongly suggests that esterification of phenols to cell-wall materials is a common theme in the expression of resistance [73–75,90]. Therefore, Santiago and co-workers [91] tried to determine if a relationship exists between phenolic compounds and resistance to stalk infection by *F. graminearum* monitoring field-grown inbred lines of maize after artificial inoculation. Reinforcing the possible role of DFAs in fungal resistance, significant negative correlations between DFAs contents and disease severity ratings four days after inoculation were found. The abundance of DFAs in cell walls makes polysaccharides less sensitive to the cell wall-degrading enzymes of pathogens [92,93]. In this case, the DFAs could function in the pith tissues as preformed resistance barriers prior to infection. Low contents of DFAs at early stages of infection would leave time for the pathogen to develop abundantly and to produce toxins, breaking a possible resistance response and leading to typical symptoms of disease.

## 5. Conclusions

In summary, cross-linking of plant cell wall polymers by dehydrodiferulates contributes consistently to the maize’s defence mechanisms against pests and diseases. Dehydrodiferulate cross-links are involved in the defence mechanisms against insects such as the European and Mediterranean corn borer, the southwestern corn borer, the sugarcane borer, and the maize weevil. In addition, DFA cross-links are also discussed to be involved in genetic resistance of maize to *Gibberella* ear rot and stalk rot. These compounds have been related with resistance in a wide range of maize tissues as leaf, kernel, rind, or pith. The role of dehydrodiferulates in the cell wall fortification has been noted in the literature, and this structural resistance could be more difficult to overcome than an antibiotic defence. Resistance against insects and fungi due to dehydrodiferulates content could go hand in hand. Quantitative trait loci mapping for these cell wall components could be a useful tool for enhancing resistance to pest and diseases in the future.

## Abbreviations:

DFA: dehydrodiferulic acid
ECB: european corn borer
MCB: mediterranean corn borer
MW: maize weevil
DON: deoxynivalenol
